# A Comparative Evaluation of Hearing and Psychological Distress in Smokers and Non-smokers: A Cross-Sectional Study

**DOI:** 10.7759/cureus.83111

**Published:** 2025-04-28

**Authors:** Khushi Meghani, Shaila Sidam, Ashish Pakhre, Aparna G Chavan, Vivek K Bharti, Anjan K Sahoo

**Affiliations:** 1 Otolaryngology - Head and Neck Surgery, All India Institute of Medical Sciences, Bhopal, Bhopal, IND; 2 Psychiatry, All India Institute of Medical Sciences, Bhopal, Bhopal, IND

**Keywords:** anxiety, depression, hearing, smoking, stress

## Abstract

Background

Smoking is a major global health concern, linked to numerous medical conditions, including hearing loss and psychological distress. Research suggests that smokers have a significantly higher risk of hearing impairment than non-smokers, possibly due to oxidative stress and vascular damage. Smoking is also associated with psychological effects such as depression, anxiety, and stress, though the relationship is complex. This study aims to compare the prevalence and degree of hearing loss between smokers and non-smokers using pure tone audiometry, and to evaluate the levels of psychological distress (depression, anxiety, and stress) in both groups using the Depression, Anxiety, and Stress Scale-21 (DASS-21) score. It also aims to assess the relationship between smoking frequency and duration with hearing loss and psychological distress.

Materials and methods

A prospective cross-sectional study was conducted at the ENT Outpatient Department of AIIMS Bhopal over three months. A total of 100 male participants aged 18-55 years were divided into two groups: 50 smokers (current or past) and 50 age- and gender-matched non-smokers. Hearing was evaluated through pure tone audiometry, tuning fork tests, and otoscopy. Psychological distress was assessed using the DASS-21, while nicotine dependence was measured using the Fagerström Test for Nicotine Dependence. Data were analyzed using IBM SPSS Statistics for Windows, Version 21 (Released 2012; IBM Corp., Armonk, NY, USA), applying the Chi-square test, with a significance level of p < 0.05.

Results

Hearing loss was significantly more prevalent in smokers (18, or 36%) than in non-smokers (7, or 14%) (p = 0.017). However, no statistically significant correlation was found between the severity of hearing loss and the frequency or duration of smoking. Psychological distress was observed in both groups, with depression (10 (20%) vs. 6 (12%)), anxiety (18 (36%) vs. 23 (46%)), and stress (2 (4%) vs. 0%) being more common in smokers, though these differences were not statistically significant. Nicotine dependence varied, with 22 (44%) of smokers having very low dependence, while two (4%) had very high dependence. Higher cigarette consumption was significantly associated with greater nicotine dependence (p < 0.001).

Conclusion

Smoking is associated with a significantly higher risk of hearing loss, reinforcing the need for awareness and early screening among smokers. However, no strong link was found between smoking and psychological distress in this study. Given the limitations of sample size and study duration, further research is needed to explore the long-term effects of smoking on both hearing and mental health. Smoking cessation programs should incorporate regular hearing assessments and psychological support for better overall well-being.

## Introduction

Cigarette smoking is a public health threat around the world, with approximately 1.3 billion people using tobacco [1]. The detrimental effects of tobacco on health are not always immediately obvious, often taking years or even decades to become apparent. Despite being the leading cause of preventable death, some measures can be taken to halt this epidemic. Nicotine, the addictive substance found in tobacco, is a compound with harmful effects and a high risk of dependence. The health consequences of smoking can vary; health may deteriorate depending on factors such as the age at which a person starts smoking, the number of cigarettes smoked per day, the intensity of inhalation, and the characteristics of the cigarettes themselves, such as their tar and nicotine content [2]. Smoking has been linked to a wide range of health issues, including lung cancer, heart disease, stroke, respiratory diseases, and many other serious conditions. It also has negative effects on overall health and well-being, including decreased physical fitness, increased risk of infection, and impaired wound healing [3].

Besides these, smoking is one of the risk factors for hearing loss and can lead to a dependent pattern [4,5]. It is estimated that smokers have a 70% higher risk of hearing impairment than non-smokers [6]. In general, smokers have 1.69 times more possibility of hearing loss compared to non-smokers [7]. The cochlea is an inner ear structure with abundant melanocytes that defend against oxidative stress. Smoking causes oxidative stress, leading to microvascular abnormalities and melanin deficiency, hence increasing the risk of hearing loss [8-10]. This is a significant finding, as it adds to the already well-established negative health effects of smoking. Hearing loss can greatly influence a person’s overall well-being, impacting their capacity to interact and engage with the world around them. 

Smoking can have various psychological effects. Nicotine in tobacco is highly addictive, leading to dependency and cravings. It can temporarily enhance mood and concentration. Smoking has been associated with an increased risk of depression, anxiety, and stress. While the relationship is complex and can vary among individuals, there is evidence suggesting a bidirectional connection. Some people may smoke as a way to cope with stress, anxiety, or negative emotions, perceiving it as a form of self-medication. On the other hand, smoking itself - particularly nicotine addiction - can contribute to changes in mood and anxiety levels. Also, smoking can influence social interactions, and smokers may face social stigma, affecting relationships and social integration.

According to a meta-analysis conducted in Japan, 9 out of 15 observational studies found a link between smoking and hearing loss [11]. Cross-sectional studies by Cruickshanks et al., Kumar et al., Sumit et al., and Chang et al. have also shown significant hearing loss associated with smoking [12-15]. Studies have shown that smoking can speed up the development of age-related hearing loss (presbycusis) [16].

Also, while there is existing research on the health effects of smoking, this study focuses specifically on how smoking impacts mental health, stress levels, social interactions, and overall well-being. This study explores psychosocial distress and hearing in smokers and non-smokers to further add critical understanding of the psychological aspects of smoking and hearing.

## Materials and methods

This prospective cross-sectional study was conducted in the Outpatient Department of ENT & Head and Neck Surgery at AIIMS Bhopal for a period of three months, from June 22, 2024, to September 27, 2024. The Institutional Ethical Committee granted ethical clearance for this study (approval no. IHEC/SR/2024/127).

A total of 100 patients were randomly selected from the Outpatient Department, and the participants were classified as smokers (i.e., past or current smokers) and non-smokers (i.e., those who have never smoked). The study was exploratory in nature, where we aimed to examine the phenomenon in two dimensions: the physical and mental domains. Thus, the sample size was derived based on the time period of data collection.

Study design

A prospective cross-sectional study was conducted. It was divided into two groups: the study group, which consisted of 50 smokers aged between 18 and 55 years, and the control group, which included 50 age- and gender-matched non-smokers, also aged between 18 and 55 years.

Inclusion criteria

The study and control groups included individuals over 18 years of age who consented to participate, and the study group included individuals with a history of smoking at least two cigarettes per day for more than six months. The control group was age- and gender-matched with the cases.

Exclusion criteria

The study did not include smokers with a history of using ototoxic drugs, any form of hearing loss, severe or recurrent ear infections, ear surgery, hypertension, or any exposure to noise. It also did not include smokers with a history of neurological disorders or any diagnosed psychiatric disorders, such as schizophrenia, bipolar disorder, etc.

The participants in the study group were selected randomly according to predefined inclusion and exclusion criteria. We used block randomization, where a block of 10 was used. Out of these 10 potential participants, we calibrated the smokers and non-smokers in a 1:1 ratio. There was no blinding, as we used block randomization. All individuals willingly took part in the study and provided informed consent before participating.

A detailed case history was taken for demographic details, and medical history, addiction, and drug history were recorded. Details regarding the duration and frequency of smoking were also noted.

Procedure

The selected cases were explained about the study, and those who consented were selected for inclusion in the study. The hearing tests were done in a soundproof room in the ENT Outpatient Department. The hearing examination included otoscopy, a screening tuning fork test, and pure tone audiometry. The tuning fork test was done using a fork with a frequency of 512 Hz. Audiometry was performed using the calibrated Maico MA 42 Puretone Portable Audiometer (Maico Diagnostics, Minneapolis, MN, USA) by an experienced audiologist who was unaware of the smoking status of the participant. The basic hearing assessment involved conducting a pure tone audiometry test, which measured hearing sensitivity across frequencies ranging from 250 to 8000 Hz.

By using the audiometric data, the hearing loss was defined as a pure tone average (PTA) of the thresholds at 500, 1000, and 2000 Hz, which were greater than the 25 dB hearing level in the worse ear. The degree of hearing loss was categorized as mild (>25 dB and ≤40 dB), moderate (>40 dB and ≤55 dB), moderately severe (>55 dB and ≤70 dB), severe (>70 dB and ≤90 dB), and profound (>90 dB) based on average pure tone results, as per the American Speech-Language-Hearing Association grading of hearing impairment [17].

All participants were interviewed using two pre-validated, self-administered questionnaires: the Depression Anxiety Stress Scale-21 (DASS-21) and the Fagerström Test for Nicotine Dependence [18,19]. The DASS-21, developed by Lovibond SH and Lovibond PF, is designed to assess three key emotional states - depression, anxiety, and stress - through self-report scales. While various tools are available for evaluating psychological well-being, the DASS-21 is notable for its superior factor loading and distinct separation of these emotional states compared to other measures. Its tripartite framework allows for a focused assessment of depression, anxiety, and stress as separate but related constructs. The questionnaire consists of three self-report scales, each addressing one of the emotional states, with seven items per scale, further divided into subscales that capture related content [18].

The Fagerström Test for Nicotine Dependence was used to determine the nicotine dependence of smokers. The reliability of this tool in predicting the success of smoking cessation attempts has been well-established. It is the most widely used quantitative measure of nicotine dependence. A higher score on the questionnaire indicates a greater level of dependence on nicotine. This tool is validated and available for free use [19]. The questionnaire was self-administered and assessed several parameters, including the number of cigarettes smoked per day, smoke inhalation, smoking habits shortly after waking up, the intention to quit smoking, smoking in restricted areas or during illness, and the urge to smoke during the first two hours of the day [19].

Statistical analysis

The statistical analysis was conducted using IBM SPSS Statistics for Windows, Version 21 (Released 2012; IBM Corp., Armonk, NY, USA). Mean and standard deviation were calculated. The Chi-square test was applied to test the significance of the degree of hearing loss in smokers and non-smokers, the relation of hearing loss with the number of bidi/cigarettes, and the duration of smoking. The Chi-square test was used to compare the DASS scores among smokers and non-smokers. A p-value <0.05 was considered significant.

Data collection and confidentiality

Data was collected using a standard proforma and questionnaire, and it was entered into a Microsoft Excel (Microsoft® Corp., Redmond, WA, USA) study database. The confidentiality of the data was maintained by allowing access to study data only to the student investigator and faculty mentor. 

## Results

In this study, the test group comprised 50 smokers, with the following age distribution: five smokers (10%) were in the 18-25 years age group, 12 smokers (24%) were in the 26-35 years range, 17 smokers (34%) were in the 36-45 years range, 14 smokers (28%) fell within the 46-55 years range, and two smokers (4%) were over 55 years of age. The control group consisted of five non-smokers (10%) in the 18-25 years age group, 13 (26%) in the 26-35 years range, 16 (32%) in the 36-45 years range, 14 (28%) in the 46-55 years range, and two non-smokers (4%) were above 55 years of age (Table 1). The mean age of smokers was 39.40 ± 10.76 years, while the mean age of non-smokers was 39.62 ± 10.40 years (Table 1). In terms of socioeconomic status, 31 (62%) smokers and 33 (66%) non-smokers lived in urban areas, and 19 (38%) smokers and 17 (34%) non-smokers lived in rural areas (Table 1). All the study participants were males. Table 1 also shows the occupations and educational status of the participants.

**Table 1:** Sociodemographic details of smokers and non-smokers Mean age ± standard deviation of smokers (39.40 ± 10.76) and non-smokers (39.62 ± 10.40).

Sociodemographic profile	Smokers (n = 50)	Non-smokers (n = 50)	Total (n = 100)	Chi-square value	p-value
Age					
18-25	5	5	10	0.104	0.917
26-35	12	13	25		
36-45	17	16	33		
46-55	14	14	28		
>55	2	2	4		
Gender					
Male	50	50	100	-	0.00
Female	0	0	0		
Location					
Urban	31	33	64	0.174	0.677
Rural	19	17	63		
Occupation					
Professionals	3	3	6	4.43	0.730
Technicians and associate professionals	3	2	5		
Clerical/admin	1	3	4		
Skilled workers and shop & market sales workers	14	18	32		
Skilled agricultural and fishery workers	16	9	25		
Craft and related trade workers	3	2	5		
Elementary occupation	7	8	15		
Unemployed/student	3	5	8		
Education					
Professional or honors degree	3	2	5	5.46	0.486
Graduate or postgraduate	13	13	26		
Intermediate or post-high school diploma	2	4	6		
High school certificate	11	18	29		
Middle school certificate	7	5	12		
Primary school certificate	4	4	8		
Illiterate	10	4	14		

As shown in Table 2, 32 (64%) out of 50 smokers exhibited no hearing loss, while 43 (86%) out of 50 non-smokers also had no hearing impairment. However, a statistically significant association between smoking and hearing loss was observed, with 18 smokers (36%) and seven non-smokers (14%) presenting with hearing impairment (p-value = 0.038). Among smokers with hearing loss, the majority had mild impairment, with 13 smokers (18%), while fewer smokers had moderate hearing loss (4 smokers, or 8%), and moderately severe hearing loss (1 smoker, or 2%).

**Table 2:** Degree of hearing loss in smokers and non-smokers using the Chi-square test *p-value < 0.05 is considered statistically significant.

Smoking status	Degree of hearing loss						Total	Chi-square value	p-value*
	Normal (upto 25 dB)	Mild (26-40 dB)	Moderate (41-55 dB)	Moderately severe (56-70 dB)	Severe (71-90 dB)	Profound (>90 dB)			
Smokers	32	13	4	1	0	0	50	8.41	0.038
Non-smokers	43	7	0	0	0	0	50		

Table 3 illustrates the relationship between hearing loss and the number of bidis/cigarettes smoked per day. The analysis revealed that the association between the severity of hearing loss and the number of bidis/cigarettes smoked daily was not statistically significant (p-value = 0.979). Furthermore, Table 3 also indicates that individuals who had been smoking for a longer duration tended to experience more severe hearing loss compared to those who had smoked for a shorter period. However, the correlation between the severity of hearing loss and the duration of smoking (in years) was not statistically significant (p-value = 0.266).

**Table 3:** Relation of hearing loss with the number of bidis/cigarettes and duration of smoking using the Chi-square test *p-value < 0.05 is considered statistically significant.

No. of bidis or cigarettes smoked per day	Degree of hearing loss						Total	Chi-square value	p-value*
	Normal (up to 25 dB)	Mild (26-40 dB)	Moderate (41-55 dB)	Moderately severe (56-70 dB)	Severe (71-90 dB)	Profound (>90 dB)			
1-10	24	10	4	1	0	0	39	2.57	0.979
11-20	3	2	0	0	0	0	5		
21-30	4	1	0	0	0	0	5		
>30	1	0	0	0	0	0	1		
Total	32	13	4	1	0	0	50		
Duration of smoking (in years)									
<5	11	3	1	0	0	0	15	14.6	0.266
5-10	8	3	0	1	0	0	12		
11-20	13	4	3	0	0	0	20		
21-30	0	2	0	0	0	0	2		
>30	0	1	0	0	0	0	1		
Total	32	13	4	1	0	0	50		

In the assessment of psychological well-being using the DASS-21 questionnaire, depression was observed in 10 (20%) smokers and 6 (12%) non-smokers. The majority of smokers reported experiencing moderate depression. However, the difference in depression rates between smokers and non-smokers was not statistically significant (p-value = 0.250) (Table 4). Anxiety was observed in 18 (36%) smokers and 23 (46%) non-smokers, with most smokers reporting moderate anxiety, while non-smokers exhibited both mild and moderate anxiety. The difference in anxiety levels between smokers and non-smokers was not statistically significant (p-value = 0.188) (Table 5). Regarding stress, two (4%) smokers reported experiencing stress, while no non-smokers exhibited stress symptoms. However, this difference was not statistically significant (p-value = 0.360) (Table 6). Overall, the findings suggest that smoking status, the quantity of cigarettes or bidis consumed, and the duration of smoking do not appear to significantly influence depression, anxiety, or stress levels in this sample.

**Table 4:** Severity of depression in smokers and non-smokers using the Chi-square test *p-value < 0.05 is considered statistically significant.

Smoking status	Normal	Mild	Moderate	Severe	Extremely severe	Total	Chi-square value	p-value*
Smokers	40	3	6	0	1	50	5.39	0.250
Non-smokers	44	2	2	2	0	50		

**Table 5:** Severity of anxiety in smokers and non-smokers using the Chi-square test *p-value < 0.05 is considered statistically significant.

Smoking status	Normal	Mild	Moderate	Severe	Extremely severe	Total	Chi-square value	p-value*
Smokers	32	3	13	0	2	50	4.78	0.188
Non-smokers	27	10	10	0	3	50		

**Table 6:** Severity of stress in smokers and non-smokers using the Chi-square test *p-value < 0.05 is considered statistically significant.

Smoking status	Normal	Mild	Moderate	Severe	Extremely severe	Total	Chi-square value	p-value*
Smokers	48	1	1	0	0	50	2.04	0.360
Non-smokers	50	0	0	0	0	50		

**Figure 1:**
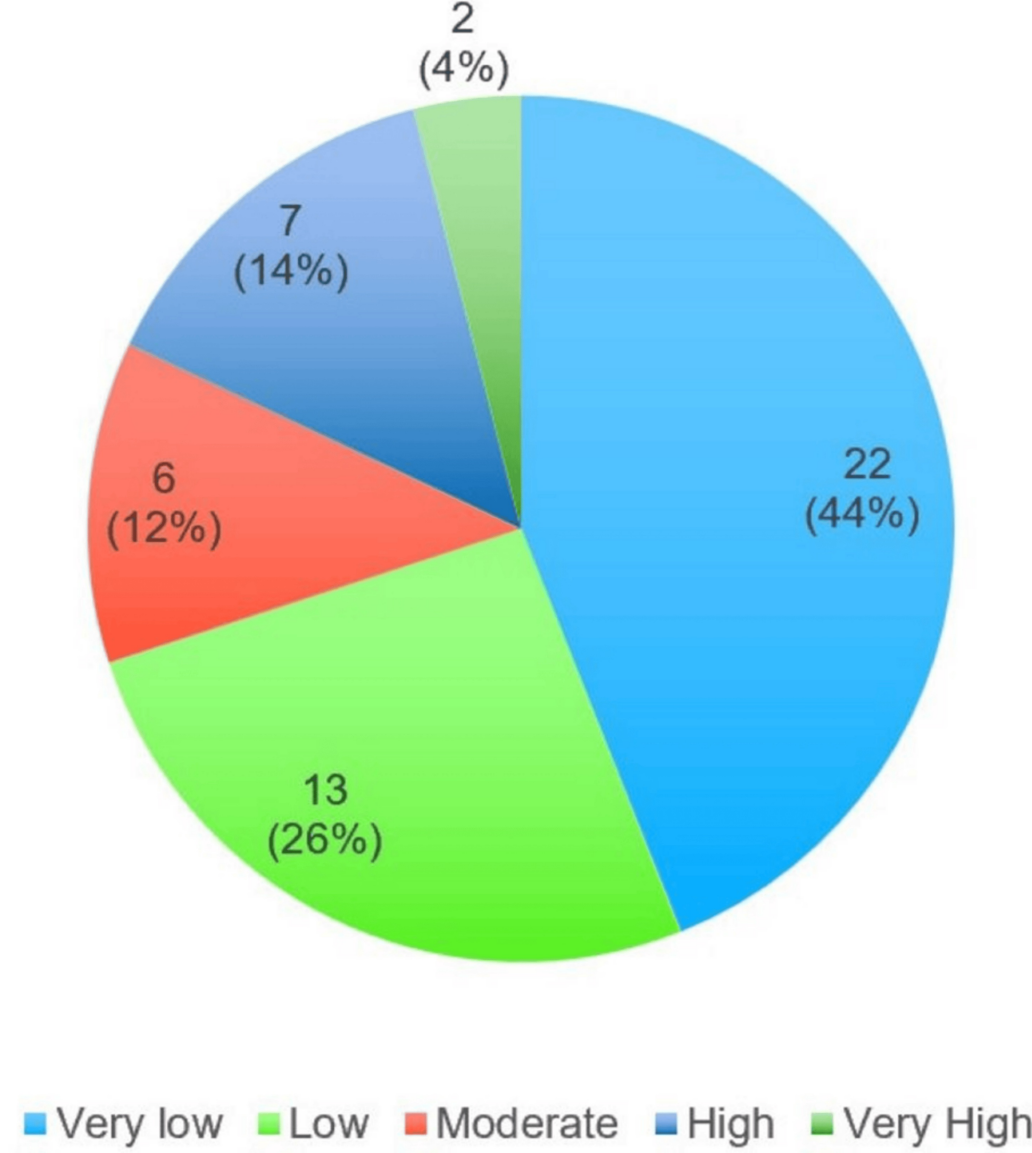
Degree of nicotine dependence among smokers

Nicotine dependence among current smokers, as assessed by the Fagerström Nicotine Dependence Scale, is presented in Figure 1. The results showed that 22 (44%) of smokers had very low nicotine dependence, 13 (26%) had low dependence, six (12%) had moderate dependence, seven (14%) were highly dependent, and only two (4%) of smokers were classified as having very high nicotine dependence.

Table 7 highlights a significant association between the frequency of smoking and the degree of nicotine dependence, with individuals who smoked a greater number of cigarettes exhibiting higher levels of dependence (p-value < 0.001). However, the table also shows that the duration of smoking was not significantly associated with nicotine dependence (p-value = 0.203).

**Table 7:** Relation of nicotine dependence with number of bidis/cigarettes and duration of smoking using the Chi-square test *p-value < 0.05 is considered statistically significant.

No. of bidis or cigarettes smoked per day	Nicotine dependence as per the Fagerström score					Total	Chi-square value	p-value*
	Very low	Low	Moderate	High	Very high			
1-10	22	11	3	3	0	39	49.4	<0.001
11-20	0	2	2	1	0	5		
21-30	0	0	1	3	1	5		
>30	0	0	0	0	1	1		
Total	22	13	6	7	2	50		
Duration of smoking (in years)								
<5	9	5	1	0	0	15	20.4	0.203
5-10	6	3	2	1	0	12		
11-20	6	4	2	6	2	20		
21-30	1	1	0	0	0	2		
>30	0	0	1	0	0	1		
Total	22	13	6	7	2	50		

Table 8 demonstrates the association of depression, anxiety, and stress with nicotine dependence. It was observed that there is no statistically significant relationship between the degree of nicotine dependence and depression, stress, and anxiety.

**Table 8:** Association of depression, anxiety, and stress by nicotine dependence categories among smokers using the Chi-square test DASS: Depression, Anxiety, and Stress Scale

DASS score	Nicotine dependence					Chi-square value	p-value
	Very low	Low	Moderate	High	Very high		
Depression							
Normal	17	10	6	5	2	4.36	0.976
Mild	1	1	0	1	0		
Moderate	3	2	0	1	0		
Severe	0	0	0	0	0		
Extremely severe	1	0	0	0	0		
Anxiety							
Normal	15	8	3	4	2	9.04	0.699
Mild	0	2	1	0	0		
Moderate	6	3	2	2	0		
Severe	0	0	0	0	0		
Extremely severe	1	0	0	1	0		
Stress							
Normal	21	13	5	7	2	8.74	0.364
Mild	0	0	1	0	0		
Moderate	1	0	0	0	0		
Severe	0	0	0	0	0		
Extremely severe	0	0	0	0	0		

## Discussion

This study follows a prospective cross-sectional design, similar to many other studies [12-15,20-22], which includes 50 smokers and 50 non-smokers, aged between 18 and 55 years. Comparable study populations were also used by Kumar et al., Sumit et al., Syed et al., and Gautam et al. [13,14,20,22]. Pure tone audiometry was conducted in this study, in line with the methodology used in other studies [20-22].

The findings of this study demonstrate a significant association between smoking and hearing loss. The relationship was statistically significant, with a p-value of 0.017, indicating that smoking is an important risk factor for hearing impairment. This result aligns with the conclusions of other studies, including Cruickshanks et al., Kumar et al., Sumit et al., Chang et al., Wang et al., and Gautam et al., all of whom have reported similar statistical correlations between smoking and hearing loss, suggesting that smoking increases the likelihood of hearing deterioration [12-15,21,22].

In this study, no statistically significant effect was found between the duration and frequency of smoking (i.e., the number of cigarettes smoked per day) and hearing loss. This may be attributed to the small sample size used in the study. However, other studies by Nomura et al., Kumar et al., Sharabi et al., Mizoue et al., Uchida et al., and Waseem et al. have shown that the prevalence of hearing loss is higher among smokers who smoke more frequently or have been smoking for a longer period [11,13,23-26]. This suggests that further research with larger sample sizes and longer smoking durations is needed to better understand the impact of smoking duration and frequency on hearing loss.

While non-smokers were also found to experience varying degrees of hearing loss, it was observed that smokers are at a significantly higher risk, with a two-fold increase in the likelihood of hearing impairment compared to non-smokers. Although this study did not explore other potential causes of hearing loss, existing research indicates that individuals exposed to secondhand smoke are nearly twice as likely to develop hearing impairment [27]. Smoking can affect hearing through several mechanisms. It increases the production of free radicals in the cochlea, disrupts nerve signal transmission, reduces oxygen supply to the organ of Corti, damages antioxidant defenses, and affects the blood vessels that supply the auditory system. The harmful substances in cigarettes, such as nicotine, carbon monoxide, and other toxic chemicals, contribute to these processes, potentially leading to hearing loss through multiple pathways [28-33].

In terms of psychological distress, the current study found that depression, anxiety, and stress were prevalent in 20%, 36%, and 4% of the smokers, and 12%, 46%, and 0% of the non-smoking group, respectively. Out of the total 100 participants in this study, depression was found in 16% of participants, anxiety in 41% of participants, and stress in 2% of them. Unlike the current findings, an Egyptian study by El-Sherbiny and Elsary found that depression, anxiety, and stress were prevalent in 41.7%, 64.2%, and 45.8% of the study participants (including both smokers and non-smokers groups), respectively [34]. An Iranian study by Fawzy et al. also showed a prevalence of depression (29%), anxiety (32.2%), and stress (34.8%) in the study participants [35]. Similarly, an Egyptian study by Al-Naggar et al., which showed that 65%, 73%, and 59.9% of the participants suffered from depression, anxiety, and stress, respectively, was also contradictory to our findings [36].

Nicotine dependence was another variable that was studied in this study. Our findings revealed that nearly half (44%) of current smokers exhibited very low nicotine dependence, as measured by the Fagerström Nicotine Dependence Scale. Additionally, 26% of smokers were categorized as having low dependence, 12% as moderately dependent, and only 14% and 4% were highly and very highly dependent, respectively. These results are consistent with those of Jayakrishnan et al., who reported that 51.5% of smokers had low dependence, 12% had moderate dependence, and 10% had high dependence [37]. Similarly, another study found 47% with low dependence, 42.5% with moderate dependence, and 10% with high dependence [38]. In contrast, the study by Shekhawat et al. observed that 24.7% of smokers had low dependence, 48% had moderate dependence, and 27.3% had high dependence [39]. A study conducted in Nepal by Aryal et al. reported that 20.4% of smokers had high dependence, and 30.3% had moderate dependence, which differs from our findings [40].

Furthermore, our study identified a significant correlation between higher nicotine dependence and smokers who consumed more than 20 cigarettes per day. This finding aligns with the study by Islam et al. [41]. These results underscore the importance of educating young people about the potential risks of nicotine dependence, as even one cigarette can lead to lifelong tobacco addiction.

Our study did not find a significant association between nicotine dependence and psychiatric symptoms, as measured by the DASS-21 score, in smokers. This contradicts previous research by Ho et al. and Branstetter et al., which found a strong association between nicotine dependence and mental health issues like stress, anxiety, and depression [42,43]. This may be because of the small sample size of the study. However, our findings are consistent with a study by Wootton et al., which also found no association between smoking and anxiety levels [44].

The limitation in this study is its small sample size and short duration of study conduction, which may account for some of the statistically insignificant results. Future research with a larger sample size would be beneficial to more accurately assess the impact of smoking duration and frequency. Another challenge encountered was the relatively low prevalence of smoking among females in India, which made it difficult to recruit female smokers in the Outpatient Department. Furthermore, this study did not consider the effects of secondhand smoke or passive smoking, which could also be an important factor in hearing loss.

## Conclusions

This study explored the effects of smoking on hearing and psychological well-being by comparing smokers and non-smokers. In conclusion, the study found that smoking harms hearing, especially as people age, with smokers showing more hearing loss than non-smokers. It also showed that smoking more cigarettes per day is linked to stronger nicotine dependence. However, there was no clear connection between smoking or nicotine dependence and levels of depression, anxiety, or stress. Based on these results, we emphasize the need for a longitudinal study with a bigger sample size, and it is recommended that smokers get regular hearing check-ups, and that smoking cessation programs include psychological support to help manage withdrawal symptoms and overall well-being.
